# The Longitudinal Role of Self-Concept Clarity and Best Friend Delinquency in Adolescent Delinquent Behavior

**DOI:** 10.1007/s10964-019-00997-1

**Published:** 2019-02-20

**Authors:** Emma K. V. Levey, Claire F. Garandeau, Wim Meeus, Susan Branje

**Affiliations:** 1 Utrecht University, Utrecht, Netherlands; 2 Tilburg University, Tilburg, Netherlands

**Keywords:** Self-concept clarity, delinquency, adolescence, longitudinal, best friend

## Abstract

Adolescence tends to be characterized by a temporary peak in delinquent behavior, and friends in particular play a key role in the initiation and the development of delinquency. However, adolescents differ in their susceptibility to friends’ influence on delinquency. Especially adolescents who are less certain about who they are might show more delinquent behavior, and might be more susceptible to their friends’ behaviors, as friends are also crucial for adolescents’ identity formation. In addition to examining the main effects of best friend’s delinquency and self-concept clarity on the development of adolescents’ delinquency, the current study scrutinized whether self-concept clarity moderated the longitudinal association between adolescents’ and their best friends’ self-reported delinquent behavior. The current study examined whether best friend delinquency and adolescent self-concept clarity were related to the development of adolescents’ delinquency, and whether self-concept clarity moderated the relation between adolescent and best friend delinquency. Dutch adolescents (*N* = 497, *M*_age_ Wave 1 = 13 years, 287 boys) and their best friends participated across six annual waves. Both adolescents and best friends reported on their delinquency and adolescents reported on their self-concept clarity. Adolescent delinquency linearly declined, and although adolescents’ and best friends' delinquency levels were related, changes in delinquency of adolescents and best friends were not. Adolescents low on self-concept clarity reported higher levels of delinquency. Self-concept clarity also moderated the relation between adolescent and best friend delinquency levels, with stronger relations observed for adolescents with lower self-concept clarity. Future research should examine the protective role of self-concept clarity not only against delinquent behavior, but also against susceptibility to peer influence.

## Introduction

On average, delinquent behavior tends to increase in early adolescence, peak in mid-adolescence, and decline in late adolescence (Moffitt 1993; Odgers et al. 2008). Most of this adolescent-limited delinquency concerns minor non-violent behaviors such as shoplifting, graffiti, and vandalism. One explanation for this temporary increase is the maturity gap: the discrepancy between adolescents’ physiological age and their lack of adult privileges or personal autonomy (Moffitt 1993). Adolescents’ individuation process from the family might be hampered by institutional influences, which causes frustration, which in turn leads adolescents to engage in behaviors that reflect independence, such as delinquent behaviors, in an attempt to express their maturity (Dijkstra et al. 2015). Deviancy might thus become a normative part of the identity search.

However, not all adolescents follow this delinquency trajectory or exhibit delinquent behaviors to the same degree (Odgers et al. 2008). Friends in particular play a key role in the initiation and the development of delinquency (e.g., Selfhout, Branje, and Meeus 2008; Slagt et al. 2015). Adolescent delinquent behavior is positively associated with the delinquent behavior of their friends, due to adolescents’ tendency to affiliate with similar others—selection effects—and to adolescents’ tendency to conform to their friends’ behaviors over time – socialization effects (Kandel 1978). However, adolescents differ in their tendency to select similar friends and their susceptibility to friends’ influence on delinquency (Müller et al. 2016; Slagt et al. 2015). Especially adolescents who are less certain about who they are might be more susceptible to their friends’ behaviors, as friends are also crucial for adolescents’ identity formation. In addition to examining the main effects of best friend’s delinquency and self-concept clarity on the development of adolescents’ delinquency, the current study examined whether self-concept clarity moderated the longitudinal association between adolescents’ and their best friends’ self-reported delinquent behavior.

### Development of Adolescent and Best Friend Delinquency

Ample research has shown that delinquency of adolescents is related to the delinquency of their friends (see Pratt et al. 2010). A number of longitudinal studies found support for associated developmental changes - suggesting that adolescents and their friends not only select each other but also affect each other’s delinquent behavior (e.g., Selfhout et al. 2008; Yu, Branje, Keijsers, Koot and Meeus 2013). For example, best friend deviant behavior was associated with higher delinquency in early adolescence (Mrug et al. 2014) and also predicted future adolescent delinquent behaviors (Rees and Pogarsky 2011; Yu et al. 2013).

Socialization effects are thought to occur because conforming to friend behavior is both extrinsically and intrinsically rewarding for adolescents (Brechwald and Prinstein 2011). Social learning theories emphasize that by modelling their friends’ behavior, adolescents engage in behaviors that are directly reinforced by friends and are therefore socially rewarding (Akers 1998). Social identity theories suggest that mimicking friends’ delinquent behaviors is intrinsically rewarding as it can elicit support and acceptance, thereby contributing to a favorable self-identity (Leary and Baumeister 2000). During adolescence, friends increasingly are primary sources of support that provide feedback and acceptance, which serves as a base for a sense of self. Therefore, best friend delinquency was expected to be positively related to adolescent delinquency.

### Self-Concept Clarity and Adolescent Delinquency

Personal characteristics can also play a role in the development of adolescent delinquency. This paper examined the role of self-concept clarity, which concerns how sure a person is of oneself (Schwartz et al. 2011). Self-concept clarity reflects the extent to which self-beliefs are clearly and confidently defined, internally consistent, and temporally stable (Campbell et al. 1996). Self-concept clarity refers to the structural aspects of the self-concept (i.e., how the self-concept is organized and integrated in memory; McConnell and Strain 2007) and it is distinct from but related to content dimensions of the self-concept. For example, self-concept clarity is positively related to self-esteem and negatively related to self-reflection (e.g., Campbell et al. 1996). Self-concept clarity is related to identity (Campbell et al. 1996; Schwartz et al. 2011), as higher self-concept clarity has been linked to stronger identity commitments (Schwartz et al. 2011). Adolescent girls tend to have lower levels of self-concept clarity than boys, and whereas adolescent girls seem to have stable levels of self-concept clarity across adolescence; adolescent boys report a slight increase from age 13 to 17 with a minor decrease thereafter (Crocetti et al. 2016). Self-concept clarity is also related to several indices of psychosocial functioning, such as anxiety and depression (Schwartz et al. 2011; Van Dijk et al. 2014).

Although links between adolescent self-concept clarity and delinquency have not been directly examined, research on identity development and adjustment suggests that questioning and rethinking one’s sense of self is associated with higher delinquency. Weaker identity commitments are related to higher delinquency (Meeus, Van de Schoot, Keijsers, and Branje 2012) and higher reconsideration of identity commitments has been related to both higher self-reported delinquency (Crocetti, Rubini, Luyckx, and Meeus 2008; Mercer, Crocetti, Branje, van Lier and Meeus 2017) and more externalizing problems (Hatano, Sugimura and Crocetti 2016). Further, young people who cannot vividly envision their future self are more likely to make delinquent choices (van Gelder, Hershfield and Nordgren 2013).

The link between identity and delinquency may be the result of developmental ambiguity and challenges (e.g., the maturity gap), if adolescents use delinquency as a means to explore their potential (self-) identity. Engaging in delinquency may also be a more general marker for difficulties in forming personal identity, a struggle which has been related to poorer adjustment in general (e.g., Meeus et al. 2012). Delinquency has been theorized to be a way of dealing with a negative self-concept (Levy 1997), and a more positive self-concept has been linked to lower assaultive delinquency (Bynum and Weiner 2002). Based on research on identity and delinquency as well as the link between self-concept clarity and identity, self-concept clarity was expected to be negatively related to delinquency.

### The Moderating Role of Self Concept Clarity

Furthermore, some adolescents are more susceptible to the influence of delinquent friends than others (e.g., Yu et al. 2013). In order to prevent and reduce youth delinquency, it is essential to identify factors that make adolescents more or less susceptible to their friends’ influence (Brechwald and Prinstein 2011). Research has found that more popular peers (Cohen and Prinstein 2006) and friends with a resilient or over-controlling personality (Yu et al. 2013) exert more influence on adolescent delinquency. Adolescent parental attachment and childhood disruptiveness also moderate the link between adolescent and best friend delinquency (Vitaro et al. 2000). A number of individual characteristics such as being male (Müller et al. 2016), having low conscientiousness (Slagt et al. 2015), low self-regulation (Gardner, Dishion, and Connell 2008), low autonomy (Allen, Chango, Szwedo, Schad, and Marston 2012) and high disinhibition (Goodnight, Bates, Newman, Dodge and Petit 2006) also have been found to serve as risk factors for increased susceptibility to influence.

The current study examined self-concept clarity as moderator of the relation between adolescents’ and their best friends’ delinquency. A less certain self-concept is hypothesized to be related to increased susceptibility to external cues (Campbell 1990), and stronger identity commitments are theorized to be related to lower susceptibility to socialization effects (Brechwald and Prinstein 2011). Moreover, adolescents without identity commitments, or without motivation to explore new commitments, are thought to be more likely to conform to peer influence due to a lack of strong beliefs of their own (Para 2008). Therefore, adolescents’ self-concept clarity is expected to affect the degree to which they are influenced by the delinquency of their friends, in that delinquency in adolescents with low self-concept clarity will be more strongly related to their best friends’ delinquency than delinquency in adolescents with high self-concept clarity.

Self-concept clarity might affect the relation between adolescent and friend delinquency differentially across adolescence. Developmental changes in susceptibility to peers indirectly suggest that self-concept clarity may play a role in this susceptibility. Research has shown a curvilinear development for conformity to antisocial peer influence (Brown et al. 1986). Increased conformity to peers is observed in early adolescence only (Shulman, Laursen, Kalman and Karpovsky 1997). Resistance to antisocial peer influence increases across adolescence, with strongest growth in mid adolescence as compared to early and late adolescence (Steinberg and Monahan 2007). Another study showed a linear increase in resistance to peer influence across adolescence (Sumter, Bokhorst, Steinberg, and Westenberg 2009). Therefore, the effect of friend influence on delinquency was expected to decrease with time. As self-concept clarity increases throughout adolescence (Crocetti et al. 2016; Wu, Watkins, and and Hattie 2010), the decrease in susceptibility to (delinquent) peer influence may be a function of psychosocial maturation in a broader sense (Brechwald and Prinstein 2011; Sumter et al. 2009). Taken together, this previous research led us to hypothesize that adolescents whose self-concept is unstable and not clearly defined may be at an increased risk of engaging in delinquent behaviors and may be more easily influenced by friends’ delinquent behavior.

## Current Study

The current study addressed two questions. First, using multivariate Latent Growth Curve Models it was examined whether best friend delinquency and self-concept clarity were related to adolescent delinquent behavior over time. The hypotheses were that best friend delinquency would be positively related to adolescent delinquency and the effect of best friend delinquency would decrease with time. Moreover, self-concept clarity was expected to be negatively related to adolescent delinquency. Second, using multigroup Latent Growth Curve Models it was investigated whether self-concept clarity moderated the longitudinal association between adolescent and best friend self-reported delinquent behavior. The hypothesis was that delinquency in adolescents with low self-concept clarity would be more strongly related to best friend delinquency in comparison to adolescents with high self-concept clarity.

## Methods

### Participants

The current study used the first six annual waves of data from 497 Dutch adolescents (56.9% boys) and their best friends, who participated in the ongoing Research on Adolescents and Relationships-Younger cohort (RADAR-Y) project (Van Lier et al. 2008). At Wave 1, the target adolescents were attending secondary school and had a mean age of 13 (*SD* = 0.46). Most adolescents lived with both biological parents (85%), were classified as having a medium to high socioeconomic status (89%) based on their caregivers’ occupations, and reported their ethnicity to be Dutch-Caucasian (95%). At Wave 6, 372 adolescents still participated in the study. Best friends were on average 13-years-old at Wave 1, *SD* = 0.80, with 49.7% of them being male. Across waves, 78.6% of friendships were same-sex friendships. Adolescents did not always have a participating best friend at each wave (17.3%), and 18 adolescents did not have a participating best friend at any wave (3.6%). Further, 28.8% (*n* = 143) of adolescents reported having the same best friend at each wave.

Attrition analyses showed no significant difference on Wave 1 delinquency between adolescents who consistently participated (*n* = 372) and those who did not (*n* = 125), *F*(1, 465) = 1.47, *p* = .226, *η*^2^ = 0.00. However, those who did not consistently participate had significantly lower self-concept clarity at Wave 1, *F*(1, 465) = 6.22, *p* = .013, *η*^2^ = 0.01, *M* = 3.29, *SD* = 0.65, compared to those who did, *M* = 3.46, *SD* = 0.63. Furthermore, non-consistent participants were slightly older at Wave 1, *F*(1, 465) = 7.76, *p* = .006, *η*^2^ = 0.02, *M* = 13.13, *SD* = 0.55, than consistent participants, *M* = 13.00, *SD* = 0.43. Attrition was not significantly related to gender, *χ*²(1) = 0.06, *p* = .806, ϕ = 0.03. Moreover, there were no significant differences on Wave 1 adolescent delinquency, *F*(1, 465) = 1.54, *p* = .215, *η*^2^ = 0.00, nor self-concept clarity, *F*(1, 465) = 2.95, *p* = .086, *η*^2^ = 0.01, between adolescents with a best friend participating at least once (*n* = 479) and those without (*n* = 18). Although Little’s test for missing data was significant, *χ*²(1536) = 1931.85, *p* < .001, ϕ = 1.97, Little’s test revealed a low χ²/*df* value of 1.26, showing a good fit of sample scores with or without using imputation methods (Bollen 1989). Thus, using Full Information Maximum Likelihood (FIML) within M*plus* (Muthén and Muthén 1998–2012) was possible. Consequently, analyses were conducted upon the entire sample (*N* = 497).

### Procedure

Target adolescents were recruited from 230 schools that were randomly selected from a list of elementary schools in the western and central regions of the Netherlands (Van Lier et al. 2008). Once recruited, adolescents were asked to invite a best friend to participate in the study. Parents of the adolescent and best friend received a thorough description of the study and were informed that their data would be treated with confidentiality. The target adolescent, their parents, their best friend, and their best friend’s parents all provided written active informed consent. Research assistants contacted the families to arrange a home visit (with yearly intervals), where adolescents and their best friend were administered questionnaires. Both adolescents and best friends received a monetary compensation of €15 for participating in each wave. Ethical permission was received from the University Medical Centre Utrecht ethical-medical committee.

### Measures

#### Self-reported delinquency

At each wave, adolescents and best friends completed the Dutch version of the Self-Report Delinquent Behavior questionnaire, based upon the International Self-Report Delinquency Study (Junger-Tas, Terlouw, and Klein 1994). This questionnaire consisted of 30 items including both minor (e.g., theft from home, vandalism) and serious offenses (e.g., burglary, selling hard drugs). For example, “In the last year, have you stolen a bike?” with answers being dichotomous (0 = no, 1 = yes). The sum score of all 30-items was used to create a variety scale for general delinquency as variety scales are generally preferred over frequency scales (e.g., Bendixen, Endresen, and Olweus 2003). More specifically, as minor offenses occur more frequently than serious offenses, frequency scales can result in a biased report, lower stability over time, and low internal consistency (Bendixen et al. 2003). Reliabilities ranged from Cronbach’s *α* = .76 to Cronbach’s α = .93 for adolescents, and from Cronbach’s *α* = = .82 to Cronbach’s *α* = = .89 for best friends, across six waves, and were all acceptable (Cronbach’s *α* = >.70; Field 2009).

#### Self-concept clarity

Each wave, adolescents completed a Dutch version of the Self-Concept Clarity scale (Campbell et al. 1996). Participants indicated how strongly they agreed with 12 statements on a five-point scale (1 = really disagree, to 5 = really agree). Example items include “I rarely have the feeling that different aspects of my personality conflict with each other” and “In general I have a clear image of who and what I am”. Sum scores were computed so that higher scores reflected higher levels of self-concept clarity. Scale reliability, construct validity, and criterion validity have previously been found adequate (Campbell et al. 1996). Further, research with Dutch adolescents has shown good internal consistency (Van Dijk et al. 2014). In the current sample, good scale reliabilities were found for all waves (*α* = .83 to .92).

### Analytic Strategy

#### Development of adolescent and best friend delinquency

**Fig. 1:**
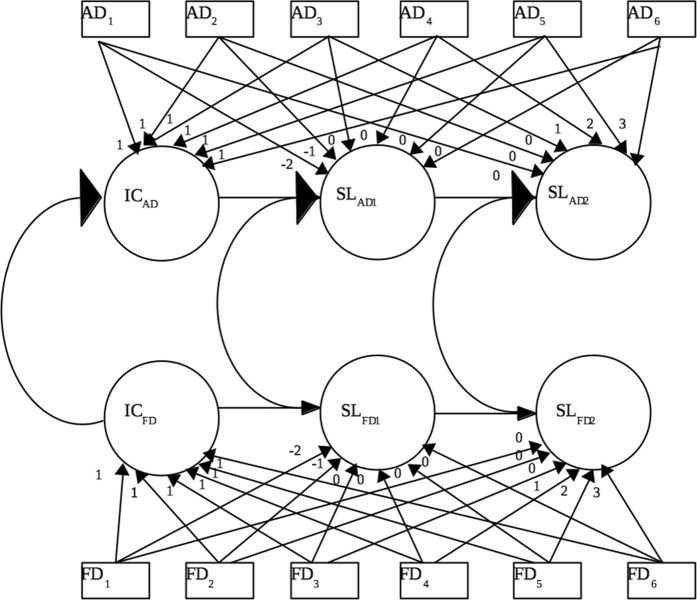
Linear growth piecewise model containing intercepts (IC_AD_, IC_FD_) slopes (SL_AD1_, SL_AD2_, SL_FD1_, SL_FD2_), and associations between adolescent _(AD)_ and friend _(FD)_ delinquent behavior

Structural equation modelling in M*plus* 7.2 (Muthén and Muthén 1998–2012) was used to conduct the analyses. Longitudinal trajectories of self-reported adolescent and best friend delinquent behavior were examined using multivariate Latent Growth Curve Models (LGCM). As data from adolescent and best friends were interdependent, the Olsen and Kenny (2006) approach, which can deal with the interdependence of data, was used. As quadratic curvilinear trajectories can be difficult to interpret and compare across multiple trajectories, piecewise latent growth analysis (Muthén and Muthén 1998–2012) was used to examine adolescent and best friend delinquency development. To fit these models, the delinquency trajectory was divided into two linear segments, with a shared intercept and two separate slopes, representing ages 13–15 and 16–18 (see Fig. 1). This cut-off was chosen as it represents the age where delinquent behavior peaks before declining (Moffitt 1993; Odgers et al. 2008).

Model fit was evaluated using the following guidelines: the Root Mean Square Error of Approximation (RMSEA) should be under .08 and .05 for acceptable and good fit respectively (MacCallum, Browne, and Sugawara 1996), the Standardized Root Mean Square Residual (SRMR) should be under .08, and the Comparative Fit Index (CFI) should be above .90 and .95 for acceptable and good fit respectively (Hu and Bentler 1999). As the delinquency data were negatively skewed, the Maximum Likelihood Ratio estimator and the Satorra and Bentler (2001) chi-square difference test (Δχ_SB_^2^) method were used to compare models.

#### Self-concept clarity and delinquency

A Latent Class Growth Analysis (LCGA) within M*plus* 7.2 (Muthén and Muthén 1998–2012) was used as a sophisticated method to distinguish groups of high and low self-concept clarity adolescents. The resulting self-concept clarity classes were used within multi-group modelling, along with Wald tests, to examine whether adolescent delinquency development differs for adolescents with either high or low self-concept Clarity. To test the moderation effect of self-concept clarity on the longitudinal relation between adolescent and best friend delinquent behavior, correlations of adolescents’ intercept with best friends’ intercept, and of adolescents’ slopes from ages 13-15 and from ages 16-18 with best friends’ slopes, were compared with univariate Wald tests.

**Fig. 2:**
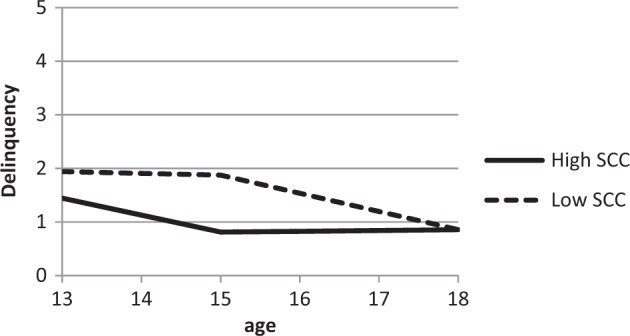
Development of adolescent delinquency per self-concept clarity group

## Results

### Distinguishing High versus Low Self-concept Clarity

The goal of this study was to examine whether adolescents with either high or low self-concept clarity show different development of delinquent behavior and differential associations with best friend delinquent behavior. Therefore, first, a Latent Class Growth Analysis (LCGA) within M*plus* 7.2 (Muthén and Muthén 1988–2012) was conducted as a sophisticated method to distinguish groups of high and low self-concept clarity adolescents.

A piecewise model allowing curvilinear change had excellent fit, *χ*^2^(12) = 28.68, *p* = .004, RMSEA = .053, CFI = .985, SRMR = .056, and fitted significantly better than a linear model, Δ*χ*_SB_^2^(4) = 42.38, *p* < .001. The two-class solution was acceptable with an entropy of .80 (>.70; Reinecke 2006), and fitted significantly better than a one-class solution, parametric bootstrapped likelihood ratio test (4) = 892.10, *p* < .001. The first class could be characterized as low self-concept clarity group (*n* = 235), with an intercept mean of 2.96, *SE* = 0.07, *p* < .001. In this group, self-concept clarity significantly decreased from ages 13–15, *M*_slope_ = −0.06, *SE* = 0.03, *p* = .044, but was stable between ages 16–18, *M*_slope_ = −0.04, *SE* = 0.04, *p* = .021. The second class could be characterized as high self-concept clarity group (*n* = 262), with an intercept mean of 3.99, *SE* = 0.06, *p* < .001. In this group, self-concept clarity significantly increased between ages 13–15, *M*_slope_ = 0.12, *SE* = 0.02, *p* < .001, but was stable from ages 16–18, *M*_slope_ = −0.00, *SE* = 0.02, *p* = .799. Class membership of self-concept clarity was significantly related to adolescent gender, with girls being more likely to be in the lower self-concept clarity group, *B* = −0.15, *SE*_B_ = 0.05, *p* = .001, *β* = −.15, but not to best friend stability as defined by adolescents reporting having the same best friends across all six years, *B* = 0.02, *SE*_B_ = 0.04, *p* = .618, *β* = .02.

### Preliminary Analyses

Correlations of adolescent and best friend delinquent behavior and self-concept clarity were examined at each wave. Adolescent and best friend delinquency were significantly moderately correlated at Waves 1 through 5, with *r*’s ranging from .23, *p* < .001 to .31, *p* < .001, but not at Wave 6 (*r* = .05, *p* = .326). Mean scores suggested that best friends were more delinquent at all waves compared to target adolescents, and that delinquency showed a linear decline across adolescence (see Table 1). Self-concept clarity was significantly but weakly related to adolescent delinquency at Waves 2 to 5, with *r‘*s ranging from −.10, *p* = .030, to −.14, *p* = .006, but not at Waves 1 (*r* = .01, *p* = .912) and 6 (*r* = −.04, *p* = .371). Furthermore, independent samples t-tests between low and high self-concept clarity adolescents (see Table 1) revealed significant differences in adolescent delinquency at Waves 2, 3, and 4, with low self-concept clarity adolescents reporting higher delinquency scores at each wave compared to high self-concept clarity adolescents.

**Table 1:** Means of adolescent delinquency and best friend delinquent behavior across waves

	Wave 1	Wave 2	Wave 3	Wave 4	Wave 5	Wave 6
	*M*(*SD*)	*M*(*SD*)	*M*(*SD*)	*M*(*SD*)	*M*(*SD*)	*M*(*SD*)
Adolescent	1.86(3.07)	1.27(2.61)	1.42(2.69)	1.28(2.65)	0.92(1.80)	0.93(2.89)
Best friend	2.35(3.08)	2.05(3.21)	2.10(3.16)	1.81(2.73)	1.74(3.07)	1.56(3.23)
Adolescent low SCC*	2.03(2.87)	1.67(3.05)^a^	1.91(3.01)^b^	1.56(2.62)^c^	1.03(1.78)	0.98(2.61)
Adolescent high SCC*	1.71(3.24)	0.92(2.10)^a^	0.99(2.29)^b^	1.03(2.66)^c^	0.82(1.81)	0.88(3.12)

Values for adolescents with high versus low SCC with the same subscript significantly differ at *p* < .05. *SCC = self-concept clarity

### Adolescent and Best Friend Delinquency Development

Development of adolescent and best friend delinquent behavior was examined using a multivariate piecewise latent growth curve model (LGCM). This model had excellent fit, χ^2^(51) = 75.10, *p* = .016, RMSEA = .031, CFI = .956, SRMR = .050, and was significantly better than a linear model, Δχ_SB_^2^(13) = 30.79, *p* = .004. A quadratic model also fitted the data well, supporting curvilinear development and the choice of a piecewise model, χ^2^(51) = 82.06, *p* = .004, RMSEA = .035, CFI = .943, SRMR = .051.

#### Testing for indistinguishable dyads

Because adolescent and best friend data are interdependent, it was tested whether adolescents and friends were indistinguishable using the Olsen and Kenny (2006) approach for modelling LGCM for interchangeable dyads. When dyad members are indistinguishable, the development of delinquency is similar for adolescents and friends. A model with latent means, latent variances, and residual variances constrained to be equal across adolescent and best friend was compared to an unconstrained model. The constrained model, in which dyad members share a common intercept, developmental pattern and developmental variance, demonstrated unacceptable fit to the data (see Model 2 - Table 2), and fitted the data significantly worse than the unconstrained model. Therefore, dyad members were not completely indistinguishable in development of delinquency. Subsequent analyses, in which paths were constrained in a stepwise procedure comparing to the unconstrained model, revealed that adolescents and best friends were distinguishable on intercept means and residual variances, but indistinguishable on slope means and intercept and slope variances (see Table 2). Thus, adolescents and best friends reported different average levels of delinquency, but similar developmental changes. The fit of this final model, with constrained slope means and variances, was excellent.

**Table 2:** Fit statistics of, and model comparisons between, models used to check for dyadic interdependence in delinquency development

Model	*χ*^2^(*df*)	*p*	RMSEA	CFI	SRMR	*Δ*χ*_SB_^2^(*df*)	*p*
Model 1: Unconstrained piecewise growth model (Baseline)	75.10(51)	.016	.031	.956	.050		
Model 2: Constrained piecewise growth model	147.98(63)	<.001	.052	.844	.107		
Model 2 vs Model 1						48.83(12)	<.001
Model 3: Intercept means constrained	87.24(52)	.002	.037	.935	.056		
Model 1 vs Model 3						23.27(1)	<.001
Model 4: Slope 1 and 2 means constrained	76.68(53)	.018	.030	.956	.050		
Model 1 vs Model 4						.34(2)	.844
Model 5: Latent variances constrained	71.62(54)	.055	.026	.968	.050		
Model 1 vs Model 5						.41(3)	.938
Model 6: Residual variances constrained	127.43(57)	<.001	.050	.871	.091		
Model 1 vs Model 6						23.01(6)	<.001
Model 7: Final model	73.26(56)	.061	.025	.968	.050		

*Chi squared difference tests were conducted using the Satorra & Bentler (2001) approach to account for the use of the MLR estimator within M*plus*.

#### Stable versus unstable friends

It was further examined whether development and associations between adolescent and best friend delinquency significantly differed for adolescents with a stable best friend across all six waves (*n* = 143) versus adolescents with unstable best friends (*n* = 354). There was no significant difference in model fit between the constrained and unconstrained model, Δ*χ*_SB_^2^(12) = 14.26, *p* = .284, and therefore it was concluded that development of delinquency in friendship dyads did not differ depending on best friend stability.

#### Delinquency development

For adolescents and best friends, delinquency levels (intercept mean) significantly differed from zero, and best friends’ delinquency level was significantly higher than adolescents’ delinquency level (see Table 3). Adolescents and best friends showed, on average, a significant decline in delinquency from ages 13–15 and from ages 16–18 (see Table 3). There was no significant difference in growth strength (slope means) between delinquency from ages 13–15 and ages 16–18, Δ*χ*_SB_^2^ (1) = .22, *p* = .64.

**Table 3:** Model parameter estimates of the final dyadic developmental model of delinquency

Model parameters	Adolescent		Best friend	
	*M(SE)*	*p*	*M(SE)*	*p*
Latent means				
Intercept	1.39^a^(0.11)	<.001	2.12^a^(0.12)	<.001
Slope 1^b^	−0.15(0.06)	.007	−0.15(0.08)	.007
Slope 2^c^	−0.19(0.04)	<.001	−0.19(0.04)	<.001
Latent variances	σ^2^(*SE*)	*p*	σ^2^(*SE*)	*p*
Intercept variance	5.32(0.86)	<.001	5.32(0.86)	<.001
Slope 1^b^ variance	1.15(0.33)	<.001	1.15(0.33)	<.001
Slope 2^c^ variance	0.54(0.15)	<.001	0.54(0.15)	<.001
Correlations	*r*	*p*	*r*	*p*
Intercept with slope 1	.36	.030	.30	.013
Intercept with slope 2	−.79	<.001	−.57	<.001
Slope 1 with slope 2	−.24	.318	.03	.871

^a^These two values significantly differ from each other at *p* < .001

^b^Slope for waves one till three

^c^Slope for waves three till six

For both adolescents and best friends, intercepts were moderately significantly positively correlated with the slope from Waves 1 till 3, but negatively with the slope from Waves 4 till 6 (see Table 3). Thus, for both adolescents and best friends, a higher delinquency level was related to a less steep decline in delinquency from ages 13-15, but to a steeper decline between ages 16 till 18.

#### Associations between adolescents’ and best friends’ delinquency

The intercept of best friend delinquency was moderately significantly positively correlated with the intercept of adolescent delinquency, *r* = .38, *p* < .001, showing that higher levels of best friends’ delinquency were related to higher levels of adolescent delinquency. However, there was no significant relation between adolescents’ and best friends’ growth from ages 13-15, *r* = .24, *p* = .170, and ages 16-18, *r* = .29, *p* = .137.

### The Moderating Role of Self-Concept Clarity

#### Self-concept clarity and adolescent delinquency

As the goal of the current study was to test whether adolescent delinquency developed differentially for adolescents with low versus high self-concept clarity, a multigroup LCGM for low and high self-concept clarity groups was fit based on the previous dyadic model, with different latent means and residual variances, but similar latent variances for adolescents and best friends. As gender was significantly related to self-concept clarity class, gender was regressed on the growth factors of delinquency to control for gender. Model fit of this baseline model was good, *χ*^2^(125) = 191.02, *p* < .001, RMSEA = .047, CFI = .923, SRMR = .076. Table 4 shows model coefficients for adolescent and best friend delinquency development per self-concept clarity group (see also Fig. 2).

**Table 4:** Model parameter estimates of the final multigroup delinquency developmental model

Model parameters	Adolescents		Best friends	
Low self-concept clarity				
Latent means	*M(SE)*	*p*	*M(SE)*	*p*
Intercept	3.05(0.59)^a^	<.001	4.10(0.68)	<.001
Slope 1^b^	−0.04(0.29)	.881	−0.04(0.29)	.881
Slope 2^c^	−0.32 (0.21)	.134	−0.32 (0.21)	.134
Latent variances	σ^2^(*SE*)	*p*	σ^2^(*SE*)	*p*
Intercept	6.48(1.18)	<.001	6.48(1.18)	<.001
Slope 1^b^	1.54(0.46)	.001	1.54(0.46)	.001
Slope 2^c^	0.62(0.18)	<.001	0.62(0.18)	<.001
Correlations	*r*	*p*	*r*	*p*
Intercept with slope 1	.64	<.001	.38	.008
Intercept with slope 2	−.91	<.001	−.62	.001
Slope 1 with slope 2	−.62	<.001	.09	.734

High self-concept clarity				
Latent means	*M(SE)*	*p*	*M(SE)*	*p*
Intercept	1.67(0.34)^a^	<.001	1.95(0.19)	<.001
Slope 1^b^	-0.46(0.23)	.044	-0.46(0.23)	.044
Slope 2^c^	0.13(0.15)	.377	0.13(0.15)	.377
Latent variances	σ^2^(*SE*)	*p*	σ^2^(*SE*)	*p*
Intercept	3.33(1.14)	.004	3.33(1.14)	.004
Slope 1^b^	0.72(0.44)	.102	0.72(0.44)	.102
Slope 2^c^	0.37(0.21)	.074	0.37(0.21)	.074
Correlations	*r*	*p*	*r*	*p*
Intercept with Slope 1	−.23	.626	.08	.782
Intercept with Slope 2	−.58	.003	−.53	.009
Slope 1 with Slope 2	.50	.421	.14	.756

^a^These paths significantly differ across groups at *p* < .010

^b^Slope for waves one till three

^c^Slope for waves three till six

Wald tests were used to examine differences in intercepts and slopes of adolescent delinquency in the low and high self-concept clarity groups. The intercept of delinquency significantly differed between self-concept clarity groups, *χ*^2^(1) = 4.15, *p* = .042. More specifically, high self-concept clarity adolescents had a lower delinquency level compared to low self-concept clarity adolescents. Although adolescents with low self-concept clarity showed stability in delinquency from ages 13-15 and adolescents with high self-concept clarity significantly declined, the slopes of the two groups did not significantly differ, *χ*^2^(1) = 1.29, *p* = .257. Similarly, there was no significant difference between the slopes of the two groups from the ages 16-18, *χ*^2^(1) = 3.00, *p* = .083.

#### Self-concept clarity and adolescents’ susceptibility to friends

To test the moderation effect of self-concept clarity on the longitudinal relation between adolescent and best friend delinquent behavior, correlations of adolescents’ intercept with best friends’ intercept, and of adolescents’ slope from ages 13-15 and from ages 16-18 with the best friends’ slope, were compared with three univariate Wald tests. There was a significant moderation effect of self-concept clarity on the correlation between adolescents’ and best friends’ delinquency intercepts, *χ*^2^(1) = 4.038, *p* = .045. More specifically, for low self-concept clarity adolescents, the correlation between adolescents’ and friends’ intercept of delinquency was significantly stronger than for high self-concept clarity adolescents. The delinquency intercept of low self-concept clarity adolescents was significantly and positively correlated with friends’ intercept of delinquency, *r* = .40, *p* = < .001, whereas this correlation was not significant for high self-concept clarity adolescents, *r* = .25, *p* = .066. However, there was no significant moderation effect on the relation between adolescent and best friend developmental slope between ages 13–15, *χ*^2^(1) = 0.06, *p* = .806, and between ages 16-18, *χ*^2^(1) = 0.04, *p* = .838. For both adolescents with low and adolescents with high self-concept clarity, the slopes were not significantly correlated, for adolescents with low self-concept clarity *r*_ages 13–15_ = .22, *p* = .257, and *r*_ages 16–18_ = .20, *p* = .486, and for adolescents with high self-concept clarity *r*_ages 13–15_ = .61, *p* = .114, and *r*_ages 16–18_ = .45, *p* = .102.

## Discussion

Delinquent behavior tends to peak in adolescence (Moffitt 1993; Odgers et al. 2008), and friends are thought to play a key role in the initiation and the development of delinquency (e.g., Selfhout et al. 2008). However, not all adolescents exhibit delinquent behaviors to the same degree (Odgers et al. 2008), and adolescents differ in their tendency to select similar friends and their susceptibility to friends’ influence on delinquency (Müller et al. 2016; Slagt et al. 2015). Conforming to friend behavior is both extrinsically and intrinsically rewarding for adolescents (Brechwald and Prinstein 2011) and can contribute to a favorable self-identity (Leary and Baumeister 2000). Adolescents who are less certain about who they are—i.e., have lower self-concept clarity—might be more susceptible than others to their friends’ behaviors. The current study examined the role of best friends' delinquency and adolescents' self-concept clarity on development of adolescent delinquency, and the moderating role of self-concept clarity on the longitudinal association between adolescents' and their best friends' delinquency. Based on previous research, the expectation was that adolescent and best friend delinquency would be related, and that higher self-concept clarity would be related to lower delinquency, but also to a lower susceptibility to influence by best friends’ delinquency patterns.

Contrary to the expected curvilinear development of adolescent delinquency often characterized by an increase until the age of 15 followed by a decline in late adolescence (e.g., Moffitt 1993), in the current study delinquency declined linearly across adolescence for both adolescents and their best friends. Moreover, although adolescent and best friend delinquency levels were significantly correlated as hypothesized, their developmental changes over time were not. In line with expectations, adolescents who scored higher on self-concept clarity had lower levels of delinquency than adolescents who scored lower on self-concept clarity. Finally, the relation between adolescents’ level of delinquency (thus not the developmental changes) and their best friend’s level of delinquency was significantly moderated by self-concept clarity. The association between adolescent and best friends’ delinquency levels was stronger for low self-concept clarity adolescents. Therefore, self-concept clarity may be a protective factor not only against delinquency, but also against susceptibility to peer influence.

### Adolescent and Best Friend Delinquency Development

In line with the hypothesis, higher average best friend delinquency was related to a higher average level of adolescent delinquency. There was a moderate, stable association between adolescent and best friend delinquency over the course of adolescence. However, developmental changes in adolescent and best friend delinquency were not related. Although the finding of a significant association in delinquency levels is consistent with previous studies showing a link between adolescent and friend delinquency (Guo et al. 2015; Weerman and Smeenk 2005), the absence of a significant association between adolescent and best friend developmental changes is discordant with the literature. Several studies have found a significant link between adolescent and best friend delinquency development (Selfhout et al. 2008 (for boys); McGloin 2009; Vitaro et al. 2000).

While friends’ delinquency is a well-established predictor of adolescent delinquency (Pratt et al. 2010; Weerman and Smeenk 2005), there are some plausible explanations for why there was no relation between the development of delinquency in adolescents and their best friends. The first explanation is related to the distinction between two different types of peer influence: situational influence and socialization influence (Hoeben and Weerman 2016). Whereas the current study examined the relation between best friends’ delinquency and adolescent delinquency (e.g., tapping into socialization effects), it is plausible that situational peer influences on delinquency may play a stronger role within delinquency development than the socialization influence of best friends’ delinquent behavior. For example, time spent in unstructured socializing with peers, a robust predictor of adolescent delinquency, is thought to influence changes in delinquent behavior via processes such as reinforcement, provocation, and instigation in the immediate situation (e.g., Hoeben and Weerman 2016). In this regard, situational peer influence may be a more salient predictor of developmental change than the long-term attitudinal and socialization influences of best friends, which may be better predictors of delinquency level. Indeed, recent experimental research suggests that even brief exposure to previously unknown deviant peers can increase deviant behavior in young people (Gallupe et al. 2016; Paternoster, McGloin, Nguyen, and Thomas 2013). This provides strong causal evidence for the saliency of situational peer influences on adolescent delinquency and shows the importance of examining time spent in unsupervised, unstructured settings for understanding within-adolescent changes in delinquency development.

Further, the importance of situational peer influences on delinquent development may also account for the unexpected linear decline in delinquency reported by both adolescents and their best friends. While time spent with peers in public and unsupervised contexts has the strongest impact on adolescent delinquency (Weerman, Bernasco, Bruinsma and Pauwels 2015), recent increases in adolescents’ internet use and gaming habits (e.g., De Looze et al. 2014) may play a role in keeping adolescents inside the parental home, instead of outside where delinquent acts are generally committed. Additionally, time spent socializing on the streets and in open spaces has been shown to decline with age in adolescence (Hoeben and Weerman 2014). This decline in both delinquency and time spent in unstructured socializing is consistent with a decrease in both self-reported and registered offences among Dutch adolescents (Van der Laan and Goudriaan 2016). Overall, general changes in how adolescents seem to be spending their time and the potentially related decreases in delinquency seen in Dutch youth are not only in line with the current study’s findings, but also provide support for the need to distinguish situational effects from socialization effects when investigating peer influence on delinquency.

A second explanation for the fact that there was no relation between developmental changes in adolescent and best friend delinquency is related to considering only best friend delinquency. For instance, research has shown that delinquency of good friends and peer groups (Haynie 2002) as well as romantic partners (Lonardo, Giordano, Longmore and Manning 2009) are related to adolescent delinquency. Further, the peer proximity hypothesis argues that close friends are more influential than general distant peers (Paek 2009). While there is some evidence that more proximate contacts matter more than distal contacts (Guo et al. 2015; Payne and Cornwell 2007), other research suggests that, under some circumstances, peer groups may have a stronger influence than best friend delinquency (Rees and Pogarsky 2011). Indeed, to understand the role of peers on delinquency, multiple characteristics of the social network as a whole should be taken into consideration (Haynie 2001). Finally, it is plausible that various characteristics of peer networks and peer relationships may be differentially related to situational and socialization influence mechanisms. Together, they may be especially relevant for disentangling influences on level of delinquency versus changes in delinquency development.

### Self-Concept Clarity and Adolescent Delinquent Behavior

Consistent with the hypothesis, the results showed that adolescents with low self-concept clarity reported higher levels of delinquency. Although the difference did not reach significance, adolescents low in self-concept clarity seemed to decline in delinquency at a later age than adolescents with high self-concept clarity, which seems to reflect a catch-up effect. Certainly, high self-concept clarity adolescents may be more self-assured and thereby have a stronger ability to avoid peer influence at a younger age than adolescents who are low in self-concept clarity. This mechanism might in part account for their lower levels of, and significant decline in, delinquency.

In line with the hypothesis that personal characteristics are related to adolescent susceptibility to peer influence (e.g., Gardner et al. 2008; Goodnight et al. 2006), self-concept clarity affected the relation between adolescent and best friend delinquency. For low self-concept clarity adolescents, the correlation between adolescents’ and friends’ intercept (but not slope) of delinquency was significantly stronger than for high self-concept clarity adolescents. This finding is consistent with previous research that has suggested that low self-concept clarity adolescents are more susceptible to external effects (Campbell 1990). Relatedly, adolescents with weaker identity commitments show increased conformity as they do not have strict beliefs to follow (Para 2008). Therefore, adolescents who are low on self-concept clarity may be more susceptible to delinquent friend influences in order to gain a sense of conformity.

Further, the findings regarding self-concept clarity have implications for delinquency interventions, which could differ in effectiveness depending on adolescent self-concept clarity levels. It is possible that interventions aimed at increasing self-concept clarity may reduce delinquency levels directly or indirectly through weakening the link between adolescent and their friend's delinquent behavior. Moreover, adolescents whose self-concept clarity is heightened through interventions may be less inclined to partake in delinquent behavior exhibited by their friends as they are less likely to be influenced by their friend’s beliefs and acts. The utility of these interventions can also be inferred from research on juvenile delinquents who have been shown to have weaker identity commitments (Klimstra et al. 2011), which has been found to be related to lower self-concept clarity (Schwartz et al. 2011). Future research should certainly consider further exploring the processes underlying self-concept clarity and delinquency to better understand this relation and its potential for delinquency prevention. Finally, because high self-concept clarity is also associated with indicators of positive adjustment such as lower depression and anxiety (van Dijk et al. 2014), efforts to increase self-concept clarity in adolescents may prove to be far-reaching in promoting healthy adolescent development.

### Limitations and Future Directions

Despite the use of a longitudinal design with multi-informant data, the current study also has a number of limitations. One limitation concerns the administration of self-reports instead of official criminal records, which can lead to biased answers. Although every effort was made to ensure the confidentiality of the participants’ answers, adolescents might have felt more pressured than their best friends to respond in a socially desirable manner due to the physical closeness of their parents during survey administration (Van de Mortel 2008). This could have contributed to the finding of a lower mean level of delinquency in target adolescents in comparison to their best friends. Nevertheless, self-reports have been shown to be a valid method of collecting delinquency data (Jolliffe et al. 2003; Thornberry and Krohn 2000), as the relatively minor delinquent acts reported by the adolescents in this study is mostly not of serious criminal nature and might not be registered in official reports. Furthermore, the use of friends’ self-reports for friends’ data removed the risk of shared observer variance. Second, the sample consisted of well-adjusted adolescents from the general population who primarily engaged in acts of minor delinquency with a limited number of more seriously delinquent adolescents and best friends, and who mostly lived in families classified as medium-to-high socioeconomic status with two parents. Had there been a larger range of self-concept clarity and delinquency scores, the examined effects may have been stronger. Also, delinquent behavior is often context-specific and might be impacted by other factors, such as the family environment, the school, or the larger peer group. It will be important for future research to examine whether the effects of self-concept clarity on delinquency and susceptibility to friends’ delinquency still hold in at-risk populations, such as low-income adolescents living in violent neighborhoods. Self-concept clarity might be less influential for individuals in environments where delinquent behavior is more normative or even adaptive.

With regard to prevention and intervention strategies, no program has to our knowledge been specifically designed to improve self-concept clarity. Nevertheless, the literature on identity formation offers a few promising avenues for future research (see Schwartz, Meca, and Petrova 2017). For example, asking individuals to write about their past while encouraging them to perceive negative events as learning opportunities might help them form a clearer self-concept (see Pennebaker 1997). Moreover, reminding individuals of their mortality has been found to promote the development of a more integrated sense of self (Landau, Greenberg, Sullivan, Routledge, and Arndt 2009) by helping them see the bigger picture when it comes to their own life narrative. Future studies could test the direct effects of such approaches on adolescents’ self-concept clarity and investigate whether these effects indirectly influence their engagement in delinquent behaviors.

## Conclusion

Although delinquent behavior tends to peak in adolescence (Moffitt 1993), not all adolescents show similar levels of delinquent behavior (Odgers et al. 2008), and adolescents differ in their susceptibility to friends’ influence on delinquency (Müller et al. 2016; Slagt et al. 2015). Adolescents who have lower self-concept clarity might be more susceptible than others to their friends’ behaviors. The current study examined the role of best friends' delinquency and adolescents' self-concept clarity on development of adolescent delinquency, and the moderating role of self-concept clarity on the longitudinal association between adolescents' and their best friends' delinquency. The findings showed that delinquency levels were positively related between adolescents and their best friends, but developmental changes were not. A higher level of self-concept clarity was associated with lower delinquency in late adolescence. Additionally, self-concept clarity was a significant moderator of the relation between adolescent and best friend delinquency levels, with higher self-concept clarity reducing the association of best friend delinquency with adolescent delinquency. The results point to the importance of further disentangling mean-level from developmental change as well as the role of situational and long-term socialization influence effects in better understanding the relationship between peers and delinquency. Finally, the finding that adolescents’ delinquency is related to their self-concept clarity highlights the importance of personal characteristics as one of the most important reasons why some adolescents manage to avoid delinquency (Moffitt 1997). Future research should attempt to further investigate the potential of self-concept clarity as protective factor against both delinquency and susceptibility to peer influence.

## Data Availability

The data that support the findings of this study are available from DANS, titled: Research on adolescent development and relationships (young cohort), https://doi.org/10.17026/dans-zrb-v5wp. Restrictions apply to the availability of these data, which were used under license for the current study, and so are not publicly available. However, data are available from the authors upon reasonable request.
